# Remote Teaching in a Preclinical Phantom Course in Operative Dentistry During the COVID-19 Pandemic: Observational Case Study

**DOI:** 10.2196/25506

**Published:** 2021-05-14

**Authors:** Philipp Kanzow, Christiane Krantz-Schäfers, Michael Hülsmann

**Affiliations:** 1 Department of Preventive Dentistry, Periodontology and Cariology University Medical Center Göttingen Göttingen Germany

**Keywords:** acceptance, COVID-19, dental education, distance learning, effectiveness, e-learning, medical education, medical student, observational, screencasts, preclinical education, remote teaching

## Abstract

**Background:**

During the acute COVID-19 pandemic, physical access to the University Medical Center Göttingen was restricted for students. For the first time at our dental school, theoretical knowledge was imparted to students via asynchronous online screencasts and discussed via synchronous video meetings only.

**Objective:**

We aimed to assess the acceptance and effectiveness of distance education as a new teaching format for theoretical knowledge within the preclinical course in Operative Dentistry (sixth semester of the undergraduate dental curriculum in Germany).

**Methods:**

The phantom course comprised distance education (first phase, 11 weeks) and subsequent on-site practical demonstrations and training (second phase, 10 weeks). All theoretical knowledge was taught via online screencasts during distance education (except for the first week, 3 screencasts were uploaded per week resulting in a total of 30 screencasts). Until the end of the term, all students (N=33) were able to view the screencasts for an unlimited number of times. Theoretical knowledge was assessed in a summative examination after practical on-site teaching. Acceptance and effectiveness of the new curriculum and distance education were also measured based on an evaluation survey and students’ self-perceived learning outcome, which was compared to the outcome from the two pre–COVID-19 terms.

**Results:**

Each screencast was viewed by a mean of 24 (SD 3.3) students and accessed a mean of 5.6 (SD 1.2) times per user (ie, by students who accessed the respective screencast at least once). During distance education, the number of accesses showed a linear trend over time. During the practical training phase, screencast views declined and increased again prior to the examination. Screencasts covering topics in Cariology, Restorative Dentistry, and Preventive Dentistry were viewed by more students than screencasts covering topics in Endodontology or Periodontology (both *P*=.047). Examination items in Periodontology showed inferior results compared to the other topics (*P*<.001). Within the different topics, students’ self-perceived learning outcome did not differ from that during the pre–COVID-19 terms. Although most students agreed that the presented screencasts contributed to their learning outcome, pre–COVID-19 term students more strongly felt that lectures significantly contributed to their learning outcome (*P*=.03).

**Conclusions:**

Screencasts showed high acceptance and effectiveness among the students but were not used as a learning tool by all students. However, students who viewed the screencasts accessed each screencast more frequently than they could have attended a conventional lecture. Screencast views were mostly due to intrinsic motivation.

## Introduction

In many disciplines, including medical education, virtual learning objects (eg, video podcasts, screencasts) are frequently and successfully used to facilitate knowledge acquisition [1,2]. As opposed to medical education, education of undergraduate dental students includes both teaching of theoretical knowledge and training of physical skills. Traditionally, during the preclinical semesters, theoretical knowledge is taught in lectures utilizing a large-group setup (ie, synchronous learning) and physical skills training is provided on-site by using dental simulators or phantom heads. The need for physical skills training renders conventional distance education (DE) within undergraduate dental education difficult. As a consequence, videoconferencing and streamed video lectures were only used by a minority of undergraduate dental schools in the past [3]. However, significant advancements in technology (eg, internet bandwidth, video conferencing hardware) have occurred in recent years. Based on a recent systematic review, the use of virtual learning objects and DE in dentistry has only been assessed in a small number of studies [4]. Most studies focusing on teaching preclinical and clinical dentistry used either virtual learning objects designed for single learning objectives [5-8], video demonstrations of practical procedures, or static PowerPoint presentations [9-11]. However, DE utilizing screen-captured lectures and video demonstrations was only reported in a single course in Prosthodontics [12]. Within the evaluation survey of this promising approach, students rated screen-captured lectures as highly useful for their self-perceived learning outcome.

During the COVID-19 pandemic, medical education required several adaptations and DE was frequently utilized [13-15]. Physical distancing measures prohibited on-site teaching activities. Moreover, dental students around the world were often unable to physically access their dental schools and dental simulators or phantom heads during the acute phase of the pandemic [16-18]. As a result, new and innovative teaching concepts, especially those focusing on theoretical knowledge, within the field of DE in dentistry rapidly emerged [19-24]. Although these teaching innovations seem promising, detailed data regarding students’ acceptance and effectiveness are often missing.

At the beginning of the COVID-19 pandemic, educators at the University Medical Center Göttingen also faced a number of challenges, as physical access to the dental school was restricted for students and on-site teaching activities were suspended. Therefore, a new curriculum featuring both DE (theoretical knowledge) and postponed on-site education (physical skills) was developed. Lectures were recorded as screencasts and distributed as online asynchronous material. For the first time, theoretical knowledge was imparted to students by using asynchronous screencasts and discussed via synchronous video meetings only. Both educators and students had no prior experience with DE. Students’ acceptance and effectiveness of DE was also unknown.

Therefore, we aimed to retrospectively analyze the acceptance and effectiveness of screencasts as a new teaching format within the preclinical phantom course in Operative Dentistry (within the sixth semester of the undergraduate dental curriculum in Germany). Further objectives of the study were to assess the use of screencasts over time, link usage data with the results of the final summative examination, and assess students’ self-perceived learning outcome and compare the results to those from the two previous pre–COVID-19 terms.

## Methods

### Study Design and Participants

During the summer-term of 2020, asynchronous screencasts and synchronous video meetings were used as means of teaching theoretical knowledge within the preclinical phantom course in Operative Dentistry at the University Medical Center Göttingen. No study-related interventions were performed. Owing to the retrospective and anonymous design of this report, no formal approval was required as stated by the ethics committee of the University Medical Center Göttingen (no. 25/12/20).

A total of 33 students were enrolled in the phantom course. Due to restricted physical access to the dental school, the course started with a phase of DE (first 11 weeks). Subsequently, on-site practical demonstrations and training of physical skills were possible (10 weeks). Thus, the summer-term 2020 was extended from 14 weeks (regular length) to 21 weeks.

### DE: Theoretical Knowledge

**Figure 1:**
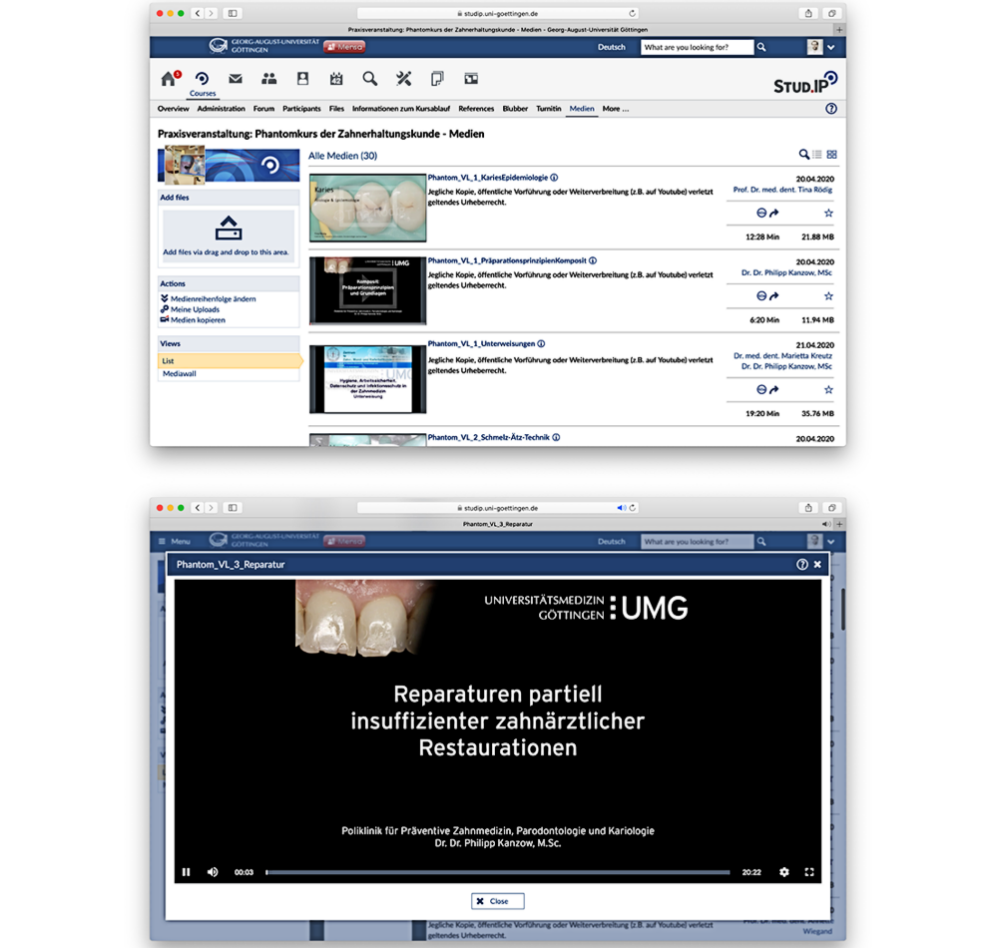
Web-based learning management system with access to screencasts. The upper panel shows the library of screencasts within the online course. Each screencast was made available via a MediaCast plugin and could be viewed using a browser-embedded media player (lower panel) or mobile devices.

All theoretical knowledge was taught via asynchronous screencasts (ie, screen-captured PowerPoint presentations with narrated audio). Starting from the second week, three screencasts were uploaded weekly, resulting in a total of 30 screencasts (Table 1). Screencasts covered three different topics: Cariology, Restorative Dentistry, and Preventive Dentistry; Endodontology; and Periodontology. Of note, the provided screencasts did not equally cover the topics. The number of screencasts per topic differed according to the relative importance of that topic and equaled the number of lectures from the pre–COVID-19 terms. Screencasts were made available to students via Stud.IP, an open-source learning management system [25], by using a MediaCast plugin (Figure 1). Anonymous data on students’ accesses to the screencasts were recorded in log files of the learning management system. Until the end of the term, students were able to view the screencasts on-demand and off-campus for an unlimited number of times. Additionally, PowerPoint presentations were available for download in PDF.

Furthermore, live and interactive video meetings (ie, Zoom videoconferencing) were offered weekly (every Thursday at 3 PM) to discuss the topics covered within the screencasts (ie, synchronous learning). Students were also able to contact their lecturers via chat (Stud.IP Blubber plugin) or forum (Stud.IP). Neither viewing of screencasts nor participation within the video meetings was mandatory.

At the end of the term, anonymous usage data were extracted from the log files to evaluate students’ accesses to the screencasts and their participation in video meetings.

**Table 1:** Characteristics of screencasts uploaded for each topic.

Characteristic	All topics	Cariology, Restorative Dentistry, and Preventive Dentistry	Endodontology	Periodontology	*P* value
Total, n (%) (N=29)^a^	29 (100)	16 (55)	9 (31)	4 (14)	N/A^b^
Duration (minutes), mean (SD)	22.9 (7.7)	18.9 (7.7)^c^	27.1 (6.0)^c^	29.8 (8.3)^c^	.02
Students who viewed screencasts, mean (SD)	24.0 (3.3)	25.5 (3.1)^c^	22.2 (2.8)^d^	21.8 (1.7)^d^	.01
Screencast accesses per user^e^, mean (SD)	5.6 (1.2)	5.6 (1.2)^c^	5.5 (1.1)^c^	5.7 (1.3)^c^	.98

^a^One mandatory screencast containing safety instructions only is not included in the presented data.

^b^N/A: not applicable.

^c,d^Different lowercase letters in a row indicate significant difference between topics after multiple-comparison posthoc correction.

^e^Students who accessed a screencast at least once were regarded as a “user” of the respective screencast.

### On-site Training of Physical Skills

In the second phase of the term, physical skills were taught on-site by using phantom heads with natural tooth models (AG-3 Frasaco) and extracted teeth embedded in resin. During this phase, physical presence of students and educators was mandatory. The students were divided into two groups to allow for sufficient physical distancing between them. Teaching hours were from 8 AM to 12:15 PM or from 12:45 PM to 5 PM on each workday (Monday through Friday). To be admitted to the final examination, students had to perform a predefined number of treatments (ie, placement of direct composite restorations and root canal treatments) with sufficient quality. Students’ work was continuously assessed by educators (experienced dentists from the Department of Preventive Dentistry, Periodontology and Cariology) present during the on-site physical skills training. For each step, students received immediate feedback.

### Electronic Examination of Theoretical Knowledge

At the end of the course, a summative electronic examination using the CAMPUS examination software (Umbrella Consortium for Assessment Networks [26]) was set. The examination consisted of 30 equally weighted items (Table 2). Single-choice items with five answer options (Type-A), multiple-select items with five or six statements (Multiple-True-False), and open-ended items were used. Single-choice and open-ended items were scored dichotomously (0 or 1 credit point per item). Multiple-True-False items were scored according to the method described by Vorkauf [27]: if all statements were marked correctly as either true or false, examinees received full credit (1 credit point). If only one statement was marked incorrectly, examinees received half-credit (0.5 credit point). Otherwise, examinees received no credit (0 credit points) [28]. A fixed pass-mark of 60% (ie, 18 credit points) was used. Again, the number of items was not equally distributed across the three topics and resembled the distribution of screencasts per topic.

**Table 2:** Characteristics of multiple-choice examination items and credit awarded to examinees for each topic.

Characteristic		All topics	Cariology, Restorative Dentistry, and Preventive Dentistry	Endodontology	Periodontology
**Items, n (%)**		30 (100)	18 (60)	8 (27)	4 (13)
	Single-choice	2 (7)	1 (50)	0 (0)	1 (50)
	Multiple-select	27 (90)	16 (59)	8 (30)	3 (11)
	Open-ended	1 (3)	1 (100)	0 (0)	0 (0)
Received credit (%), mean (SD)		74.5 (34.6)	75.8 (34.5)^a^	79.2 (31.2)^a^	58.9 (37.2)^b^

^a,b^Different lowercase letters in a row indicate significant difference between topics after multiple-comparison posthoc correction.

### Students' Self-Assessment of Learning Outcome

Immediately after the electronic examination, a standardized evaluation survey was electronically administered to all students using the EvaSys software (version 8.0; evasys). The questionnaire comprised a number of closed items and utilized a 6-point Likert scale with the following response options: 1=“totally agree,” 2=“agree,” 3=“mostly agree,” 4=“mostly disagree,” 5=“disagree,” and 6=“totally disagree.” Although the focus was primarily on organizational aspects, some items assessed students’ self-perceived learning outcome (ie, “I estimate my learning outcome in Preventive Dentistry/Restorative Dentistry/Endodontology/Periodontology as high” and “The lectures/practical training/practical demonstrations in this course significantly contributed to my learning outcome”). Students were able to provide additional information and further suggestions in a final open-ended question. For analysis of the open-ended responses, a qualitative content analysis with inductive categories regarding aspects related to DE was performed.

### Statistical Analysis

All data were first reported descriptively as absolute numbers (categorial variables) or using mean and SD values (continuous variables). Subsequently, usage data and examination results were compared between the three topics (Cariology, Restorative Dentistry, and Preventive Dentistry; Endodontology; and Periodontology) by using Kruskal-Wallis rank sum tests followed by Dunn posthoc tests. In addition, students’ self-perceived learning outcome was compared to evaluation surveys from two previous terms involving conventional lectures instead of screencasts by using Kruskal-Wallis rank sum tests followed by Dunn posthoc tests.

All statistical evaluations were performed using R software (version 4.0.3; The R Foundation for Statistical Computing) and the packages “PMCMR” (version 4.3) and “irr” (version 0.84.1). The level of significance was set at *P*<.05. Multiple-comparison posthoc correction was performed using Hochberg method.

## Results

### DE: Theoretical Knowledge

Theoretical knowledge was taught by using a total of 29 screencasts, with a mean length of 22.9 (SD 7.7) minutes. Each screencast was viewed by a mean of 24 (SD 3.3) students (range: 17-29 students). Users (ie, students who accessed the respective screencast at least once) accessed each screencast a mean of 5.6 (SD 1.2) times. Detailed results for each topic are presented in Table 1. Screencasts in Cariology, Restorative Dentistry, and Preventive Dentistry were viewed by more students (mean 25.5, SD 3.1) than screencasts in Endodontology (mean 22.2, SD 2.8) or Periodontology (mean 21.8, SD 1.7; both *P*=.047). The average number of screencast accesses per user did not differ between the topics (Cariology, Restorative Dentistry, and Preventive Dentistry: mean 5.6, SD 1.2; Endodontology: mean 5.5, SD 1.1; Periodontology: mean 5.7, SD 1.3; *P*=.98).

**Figure 2:**
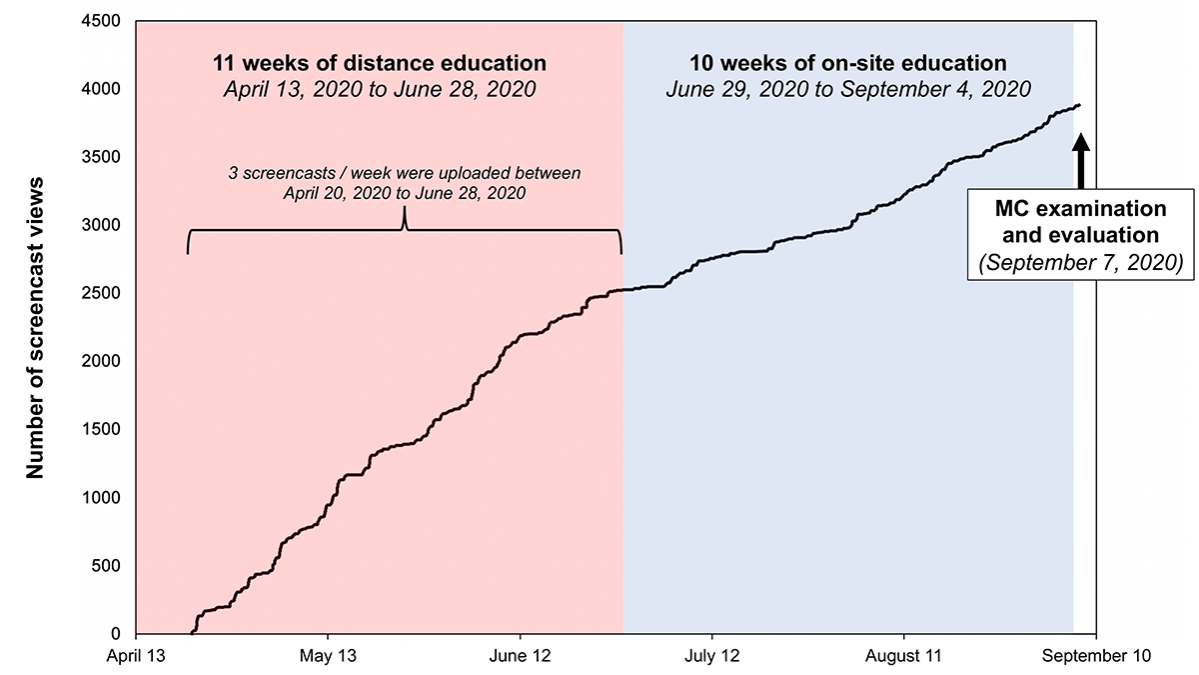
Number of screencasts views over time. Time spans of distance education (theoretical knowledge) and on-site education of physical skills are marked by different colors. All screencasts were uploaded during the distance-education phase. The final examination and evaluation were set after the on-site education phase. MC: multiple-choice.

**Figure 3:**
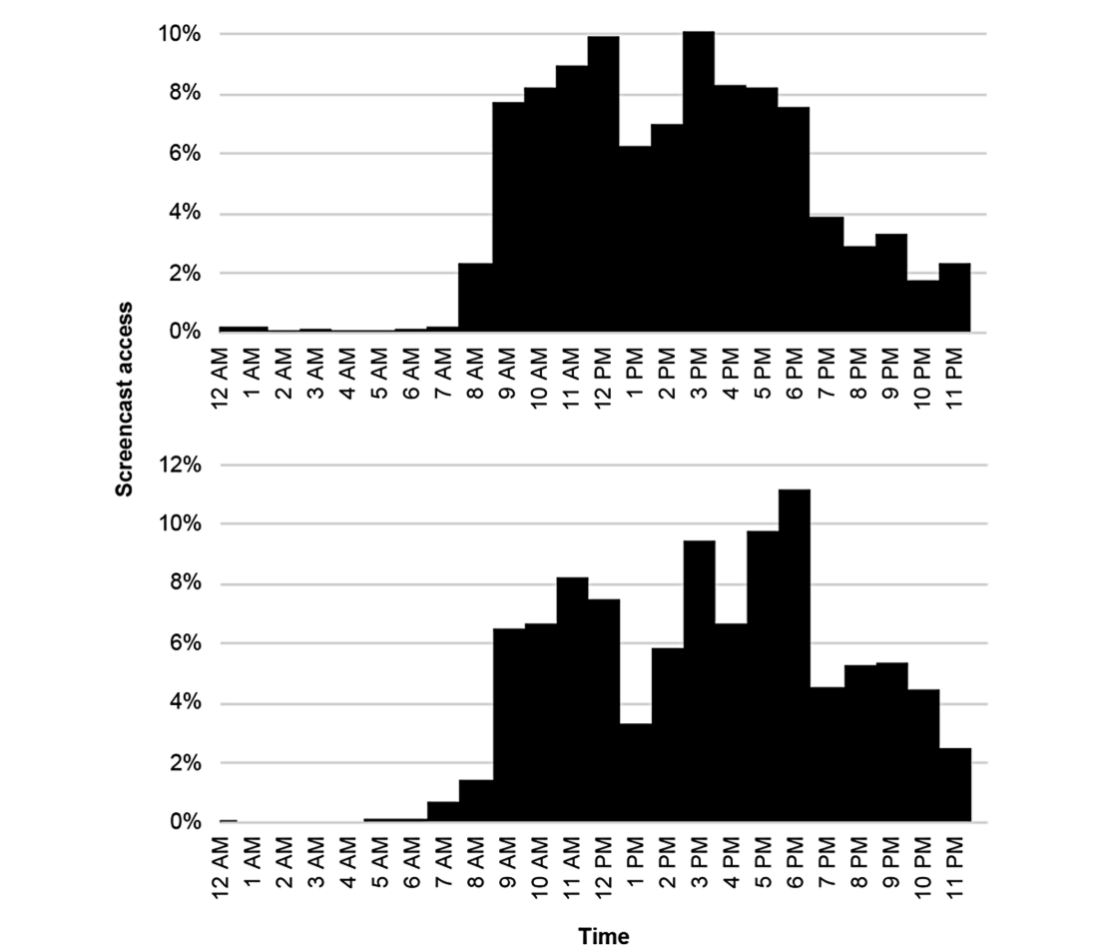
Histograms showing the distribution of screencast access times over the course of the day. The upper panel shows the access times during the distance education phase. The lower panel shows access times during the on-site phase of physical skills training. During on-site teaching, physical presence at the dental school was mandatory on weekdays (either between 8 AM to 12:15 PM or between 12:45 PM to 5 PM).

During the phase of DE, the number of screencast accesses showed a linear trend over time. The number of screencast views also declined during the subsequent practical training but increased again prior to the final examination (Figure 2). Mostly, screencasts were accessed in the morning and afternoon hours. Screencasts were also viewed in the evening hours. Around noon, fewer numbers of accesses were observed (Figure 3).

The mean number of students who participated at the live and interactive video meetings was 21.2 (SD 6.7). Weekly video meetings were held to answer students’ questions and discuss the content of screencasts (duration: mean 13.1, SD 6.3 minutes).

### Electronic Examination of Theoretical Knowledge

Only 31 students met the course requirements during physical skills training and were eligible for taking the final examination. Overall examination difficulty (ie, the mean score per item in the given situation) amounted to 0.74. Items in Periodontology showed inferior results compared to the other topics (58.9% vs 75.8% for Cariology, Restorative Dentistry, and Preventive Dentistry and 58.9% vs 79.2% for Endodontology; both *P*<.001).

### Students' Self-Assessment of Learning Outcome

Students’ self-perceived learning outcome within the assessed topics did not differ from the evaluations performed during the pre–COVID-19 terms (Restorative Dentistry: *P*≥.21, Preventive Dentistry: *P*=.84, Endodontology: *P*≥.48, and Periodontology: *P*=.36; Table 3). Regarding DE, most students agreed that the presented screencasts significantly contributed to their learning outcome (median score: 2=“agree”). However, students from the pre–COVID-19 terms rated more strongly that lectures significantly contributed to their learning outcome within the preclinical course in Operative Dentistry (*P*=.03). Evaluation of practical training during on-site teaching did not significantly differ from that during the pre–COVID-19 terms (*P*≥.69). The contribution of practical demonstrations showed comparable results to the previous phantom course during the pre–COVID-19 winter-term 2019/20 (*P*=.27) but was judged as less supportive than that during the summer-term 2019 (*P*=.03).

In response to the final open-ended question, some students gave additional insights regarding their perception of DE: students criticized the screencasts as being superficial (n=4), shorter than conventional lectures (n=2), and an inappropriate learning tool for the final examination (n=2). Some students (n=2) also criticized the need for additional self-study.

**Table 3:** Students’ self-assessment of learning outcome during summer-term 2020 and the pre–COVID-19 terms.

Survey item^a^	Summer-term 2020 (n=31, response rate: 94%)	Winter-term 2019/20 (pre–COVID-19) (n=33, response rate: 97%)	Summer-term 2019 (pre–COVID-19) (n=29, response rate: 78%)
	Median (IQR; range)	Median (IQR; range)	Median (IQR; range)
“I estimate my learning outcome in Preventive Dentistry as high.”	2 (1-2; 1-4)^b^	2 (1.25-2; 1-4)^b^	2 (1-2; 1-4)^b^
“I estimate my learning outcome in Restorative Dentistry as high.”	2 (1-2; 1-3)^b^	2 (1-2; 1-4)^b^	1 (1-2; 1-3)^b^
“I estimate my learning outcome in Endodontology as high.”	1 (1-2; 1-3)^b^	1 (1-2; 1-3)^b^	1 (1-2; 1-3)^b^
“I estimate my learning outcome in Periodontology as high.”	3.5 (3-4; 1-6)^b^	3 (2.25-3.75; 1-6)^b^	3 (2-4; 1-6)^b^
“Lectures significantly contributed to my learning outcome.”	2 (2-3; 1-5)^b^	2 (2-2; 1-4)^c^	2 (1-2.25; 1-5)^c^
“Practical training significantly contributed to my learning outcome.”	2 (1-2; 1-4)^b^	2 (1-2; 1-3)^b^	2 (1-2; 1-3)^b^
“Practical demonstrations significantly contributed to my learning outcome.”	2 (2-3; 1-3)^b^	2 (1-3; 1-5)^b,c^	1.5 (1-2; 1-3)^c^

^a^Students’ responses on a 6-point Likert scale with the following response options: 1=“totally agree,” 2=“agree,” 3=“mostly agree,” 4=“mostly disagree,” 5=“disagree,” and 6=“totally disagree.”

^b,c^For each item, different lowercase letters in a row indicate significant difference between the terms after multiple-comparison posthoc correction.

## Discussion

### Principal Findings

This study reports the experience of a German dental school with DE in a preclinical phantom course in Operative Dentistry. Due to the COVID-19 pandemic, the current curriculum had to be adapted. As further development of the pandemic was unknown, a high degree of planning uncertainty was present throughout the term. During the initial phase, feasibility of the new curriculum was still unknown. Moreover, both educators and students were not used to DE, and students’ acceptance of screencasts as a new teaching format was unknown.

### Acceptance of DE

Students’ attention in conventional lectures is known to start decreasing after only 10 minutes [29]. Regarding videos in massive open online courses, video lengths of varying durations between 6 and 20 minutes are recommended in the literature [30]. Therefore, produced screencasts were kept shorter (duration: mean 22.9, SD 7.7 minutes) than conventional lectures from the pre–COVID-19 terms (duration: 45 minutes). In addition, screencasts included references to selected articles and book chapters for further reading. Students were encouraged to review the presented topics during self-study. Weekly live and interactive video meetings were offered to discuss any questions. The number of students participating in the video meetings was slightly lower than the number of screencast users (mean 21.1, SD 6.7 vs 24.0, SD 3.3).

The term could be performed as initially planned. At the end, data on screencast usage over time were assessed and linked to examination results. Screencasts were not used by all students as a learning tool. Up to 4 students refrained from viewing at least a single screencast. However, students using the screencasts accessed each screencast more frequently than they could have visited a conventional lecture. Screencast viewing was mostly due to intrinsic motivation as screencast accesses showed a linear trend already at the beginning of the term. However, the final examination triggered an extrinsic increase in screencast accesses immediately prior to the examination date. This increase prior to the examination is in accordance with the observed access patterns in a growth and development curriculum: web-based learning modules were more frequently accessed by dental students as course examinations approached [31].

Interestingly, most screencasts were accessed during the daytime and evening hours, indicating that students seem to have maintained their daily routine during DE without any mandatory courses, as only an absolute minority of screencasts views were noted after midnight. In addition, access rates dropped around 1 PM, suggesting students took a lunch break around noontime. The pattern of access times only slightly shifted between both phases: during on-site teaching, screencasts were more frequently accessed in the evening hours. As always, only half of the cohort was present in the dental school for on-site teaching, and the other half was able to access the screencasts also in the morning or afternoon hours.

### Effectiveness of DE

This study reports on the effectiveness of DE in an undergraduate dental curriculum. Students’ acceptance and the effectiveness of DE were assessed based on the number of screencast views, students’ summative examination results, and students’ self-perceived learning outcome.

As physical attendance of lectures was not mandatory during the pre–COVID-19 terms, no comparison between the number of users and students attending conventional lectures was possible. Results of the final examination are comparable to those from the pre–COVID-19: within the phantom course, examination difficulty ranged between 0.64 and 0.82 over the past terms. However, this comparison should be interpreted with caution as examination items differed.

Some students criticized that the presented screencasts were very superficial and/or very short. However, screencasts were intentionally kept shorter than conventional lectures in the pre–COVID-19 terms for didactic reasons. Although the students’ self-perceived learning outcome did not differ from the past terms and most students agreed that the presented screencasts significantly contributed to their learning outcome, pre–COVID-19 term students rated more strongly that lectures significantly contributed to their learning outcome. Again, this comparison with students of the previous terms should be interpreted with caution, as evaluations were performed at different time points. For instance, although the evaluations of previous terms were performed near the end of the practical training, the current evaluation was performed immediately after the final examination. Therefore, the examination might have affected the students’ judgement, leading to biased evaluation results.

Overall, the acceptance of DE can be regarded as high, and most students agreed that screencasts significantly contributed to their learning outcome. The presented data show the promising use of DE in an undergraduate dental curriculum. Our results are in line with those of a previous study that found that screen-captured lectures and video demonstrations were rated as highly useful by students regarding their self-perceived learning outcome in a course in Prosthodontics [12].

### Limitations

The major limitation of this study is the anonymous data structure used. Therefore, no demographic data or other student-related factors concerning the use of the screencasts were available. In addition, no correlation of screencast viewing, examination results, or evaluation survey responses was possible at the individual student level. No data regarding the technical devices used and how students accessed the screencasts were available. Therefore, potential restrictions (eg, no device or internet access, not enough time to view screencasts) preventing some students from accessing the screencasts could not be identified. Moreover, the possibility that screencasts were jointly viewed by multiple students per access cannot be excluded.

A standardized questionnaire was used for the final evaluation survey. The evaluation survey was not modified according to the COVID-19 situation and the modified curriculum in effect (ie, DE and extended term duration). More detailed results could have been obtained by using a more differentiated questionnaire.

Further research regarding DE within the field of dentistry is required. These studies should allow for a direct comparison between screencast usage and examination results at the individual student level, assess students’ self-estimated learning outcome using more detailed questionnaire tools, and include a control group.

### Conclusions

Within the abovementioned limitations of the study, the results show that DE using online screencasts is a viable way of imparting theoretical knowledge in undergraduate dentistry programs. Screencast usage seems to be linked to examination results, and screencasts should be made available to students in addition to conventional lectures when the regular curriculum can be resumed. As suggested by some students, the length and content of screencasts could also be extended.

## Abbreviations

DE: distance education
